# A case report of a long-term abandoned torn lingual nerve injury repaired by collagen nerve graft induced by lower third molar extraction

**DOI:** 10.1186/s40902-019-0243-z

**Published:** 2019-12-23

**Authors:** Shigeyuki Fujita, Naoki Mizobata, Takashi Nakanishi, Itaru Tojyo

**Affiliations:** Department of Oral and Maxillofacial Surgery, Wakayama Medical University, 811-1 Kimiidera, Wakayama, Wakayama Prefecture 641-8509 Japan

**Keywords:** Lingual nerve, Iatrogenic injury, Mandibular third molar extraction, Allograft collagen nerve

## Abstract

**Background:**

The lingual nerve plays an important role in multiple functions, including gustatory sensation and contact sensitivity and thermosensitivity. Misdiagnosed conservative treatments for serious lingual nerve (LN) injuries can induce the patient to serious mental disability. After continuous observation and critical diagnosis of the injury, in cases involving significant disruption of lingual nerve function, microneurosurgical reconstruction of the nerve is recommended. Direct anastomosis of the torn nerve ends without tension is the recommended approach. However, in cases that present significant gaps between the injured nerve ends, nerve grafts or conduits (tubes of various materials) are employed. Recently, various reconstruction materials for peripheral nerves were commercially offered especially in the USA, but the best method and material is still unclear in the world. There currently exists no conventional protocol for managing LN neurosensory deficiency in regard to optimal methods and the timing for surgical repair. In Japan, the allograft collagen nerve for peripheral nerves reconstruction was permitted in 2017, and we tried to use this allograft nerve and got a recommendable result.

**Case presentation:**

This report is a long-term abandoned torn LN reconstructed with allograft nerve induced by the lower third molar extraction.

**Conclusions:**

In early sick period, with the exact diagnosis, the LN disturbance should be managed. In a serious condition, the reconstruction with allograft nerve is one of the recommendable methods.

## Background

Extraction of the mandibular third molars is one of the most popular procedures in oral and maxillofacial surgery. As a complication of tooth extraction, injuries of the lingual nerve (LN) may cause grave lingual sensory and taste disorders. The incidence is very rare; the paresthesia of patients is severe.

Misdiagnosed conservative treatments for serious LN injuries can induce the patient to serious mental disability. In this case, the patient had depended much upon psychotropic medicine for more than 3 years without a critical diagnosis for the disturbed LN. There currently exists no conventional protocol for managing LN neurosensory deficiency in regard to optimal methods and the timing for surgical repair. Recently, various reconstruction materials for peripheral nerves were commercially offered especially in the USA, but the best method and material is still unclear in the world. The Ministry of Health, Labor and Welfare of Japan permitted to use the allograft collagen nerve for peripheral nerve reconstruction in 2017; we tried to use this allograft nerve and got a recommendable result. The details of patients with iatrogenic LN disorders abandoned for 17 years which underwent microsurgical repair are explained.

## Case presentation

### Present history

Two years before, a 59-year-old Japanese woman was referred to our department because of a mental anxiety and intense pain in the left tongue after the extraction of the right mandibular third molar 17 years ago. To manage such a serious perception abnormality, she had received stellate ganglion block (SGB) ten times and medical treatment in various psychosomatic medicine and psychiatry and received various medication of psychotropic drugs for more than 3 years. Without any effectiveness, she suddenly had dropped in panic disorder and depression many times.

On her first visit to our Department of Oral and Maxillofacial Surgery, Wakayama Medical University, we checked the details for the tongue perception with various medical examination methods for the first time.

Our neurosensory assessments included the following.

### Subjective assessment

The subjective assessment done was the rating of the subjective sensation (visual analog scale) of the affected area, which was the most seriously bad limited right point.

### Objective assessment

1. Brush stroke directional sensation with camel hair brush (brush); horizontal, vertical, and rotational stimulating movement were applied (0 means recognized not at all, 1 means recognized only one direction, 2 means recognized two directions, and 3 means recognized all movements): the left anterior region of her torn tongue revealed 3 but the posterior region indicated 0.
2. Pinprick test: sharp touch reaction with needle, which was indicated in poor reaction.
3. Static two-point discrimination (2PD): the left anterior region reacted in 8 mm but the left posterior region reacted in 15 mm.
4. Pressure pain threshold: the Semmens-Weinstein monofilament (SWM), composed of 20 different diameter monofilaments: 1 was assigned to the smallest diameter and 20 was the largest diameter monofilament. With each instrument, the anterior region of her torn tongue reacted with 5 and the posterior region reacted with 8.
5. Thermal discrimination (thermal): hot and cold sensation; hot water 42 °C; cold sensation, ice 0 °C, pin prick. Both her thermal reactions were positive.
6. Tinel’s reaction on the torn lingual nerve: against this reaction, she received a violent sharp stabbing pain along the left side of her disturbed tongue.
7. Gustatory sensation assessed with localized testing discs (Sanwa Kagaku Kenkyusho, Japan): salty, sodium chloride 1 mol/l; sweet, sucrose 1 mol/l; sour, acetic acid 0.4 mol/l; bitter, quinine 0.1 mol/l, which were absent for all reagents on the injured side of her tongue. All the abovementioned inspections were performed at pre-operation and at 6 months after the microsurgery retrospectively. The outcome was analyzed with Sunderland grade and by the Medical Research Council Scale (MRCS). Summarizing such inspections of careful tests, we diagnosed this case as an old serious perception abnormality in the left LN. The detailed data is presented in Table 1.

**Table 1:**
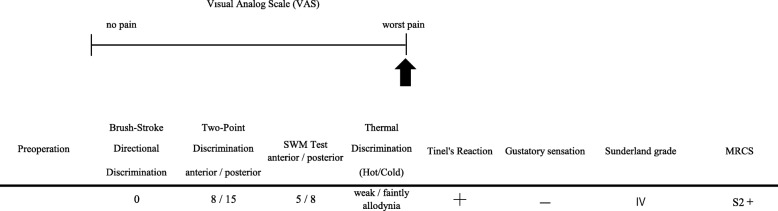
Preoperative data of visual analog scale and another various assessments

After taking informed consent with patient and family, we decided to enforce the lingual nerve reconstruction with allograft nerve microsurgically.

### Surgical procedure

**Fig. 1:**
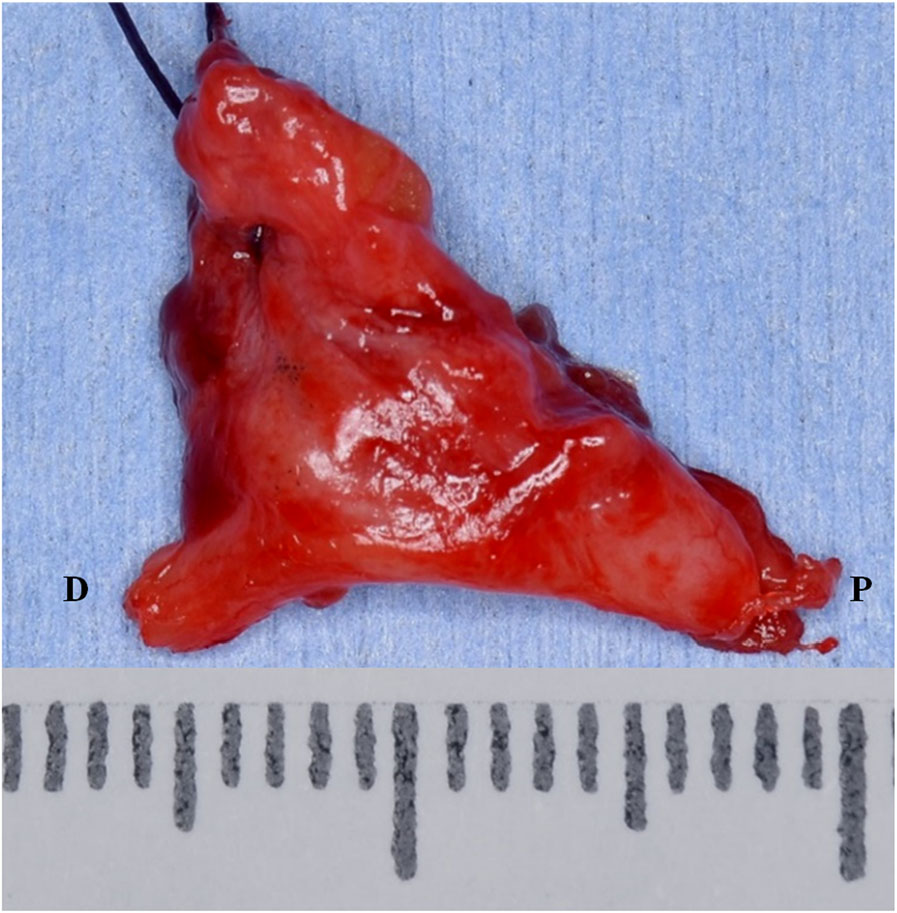
The granuloma and peripheral neuroma. The removed torn LN and scar tissue was about 14mm length

**Fig. 2:**
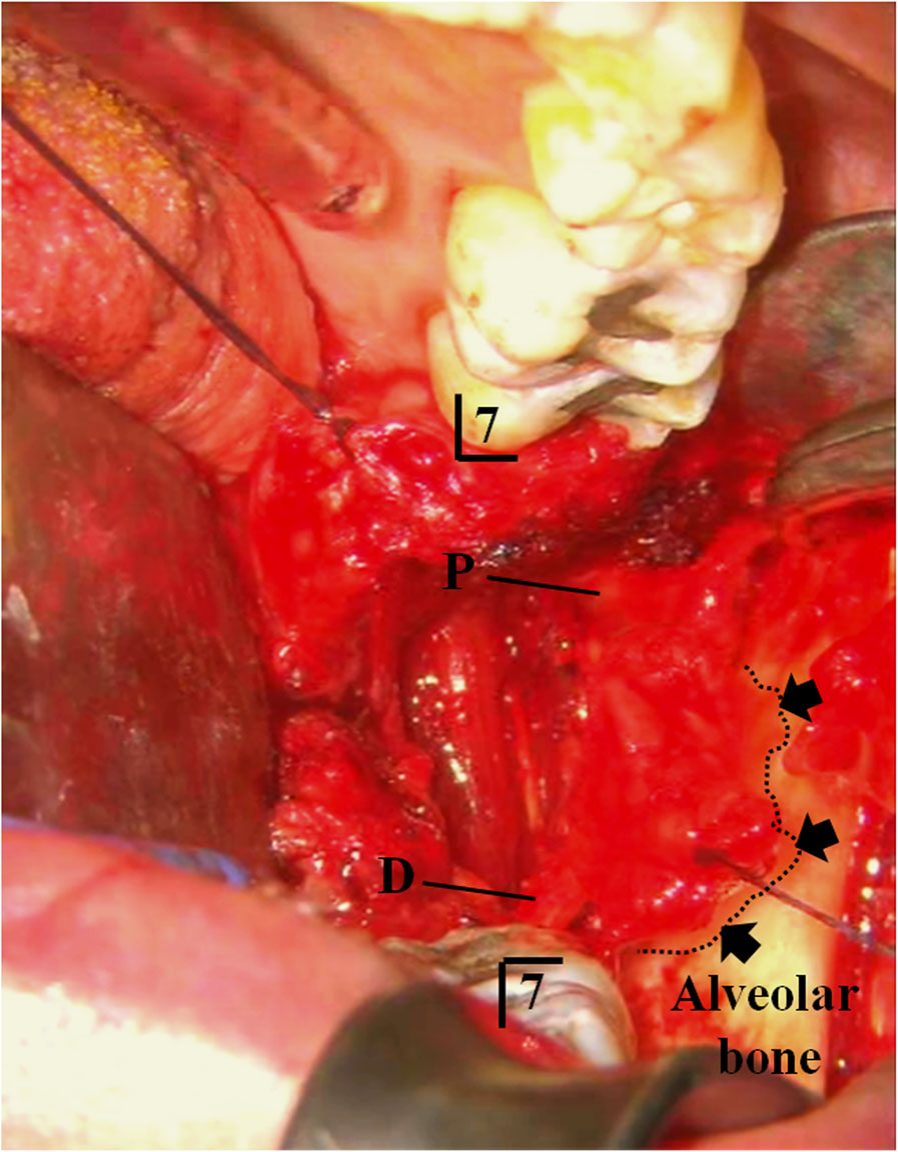
LN injury site and a large granuloma of LN. Operative findings; the torn LN and scar tissue are identified in the socket of wisdom tooth (arrow). P: Proximal end LN, D: Distal end LN

**Fig. 3:**
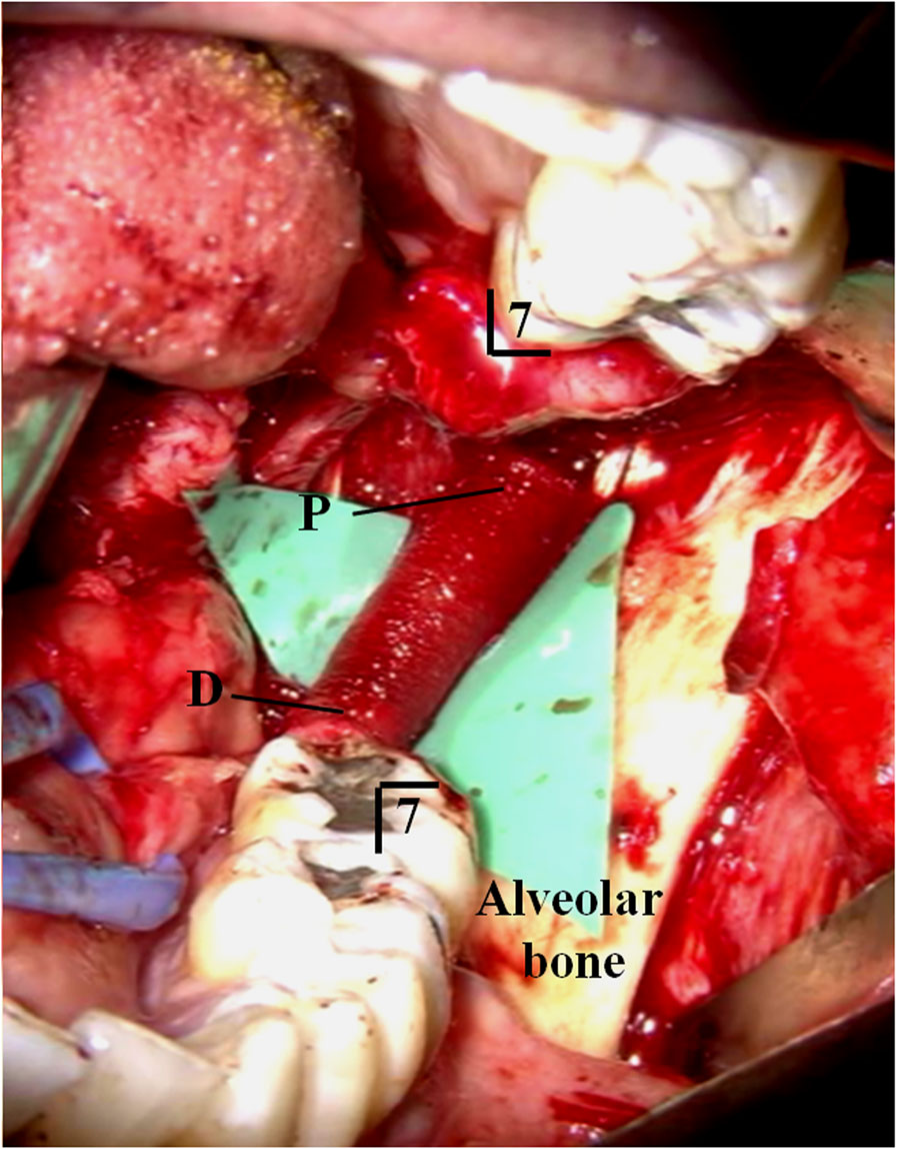
Sutured LN through allograft nerve (RENERVE®). Operative findings; Between each end of rest LN, allograft nerve (RENERVE®) about 18mm length was inserted and sutured with 8-0 nylon in the microsurgical field

Microsurgical treatment of LN injury was performed under general anesthesia. The LN was exposed through an intraoral mucosal incision. A large granuloma of LN adjacent to extracted cavity of third molar teeth was revealed. The granuloma and peripheral neuroma surrounding the torn LN about 14 mm length were removed completely (Fig. 1). Between each end of lingual nerves, allograft nerve (RENERVE®) about 18 mm length was inserted and sutured with 8-0 nylon in the microsurgical field (Figs. 2 and 3).

At 6 months and 1 year after the operation, the patient showed an amazingly improved sensory recovery reaction. The data of SWM test, 2PD, Tinel’s reaction, and gustatory sensations improved remarkably (Table 2). It is incredible 1 year later of the microneurosurgical operation; she was free from psychotropic drugs except for a sleep inducer and regained a sound healthy daily life.

**Table 2:**
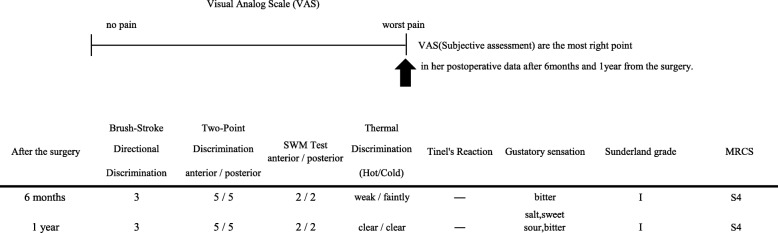
Postoperative data of visual analog scale and another various assessments

## Discussion

The main cause of LN injury is inadequate exodontia. Therefore, clinicians have to learn adequate surgical technique of the third molar and must understand the risk factors associated with injury to the LNs. The torn LN injury should recover as soon as possible with the best methods.

Slight peripheral nerve injury may be healed spontaneously without any surgical management, for example, in the phase of neurapraxia on Seddon analysis. As a common sense, in the early phase of suspicious spontaneous healing, critical examination like a clinical neurosensory testing (CNT) should be performed as soon as possible [1]. Currently, the conventional protocol for managing LN neurosensory deficiency in regard to optimal methods and the timing for surgical repair is not recognized. In the case of completely damaged case, microsurgical reconstruction should be utilized. Generally, grafting is unadvisable for LN repair because the nerve path is tortuous enough to mobilize without tension [2]. The best functional nerve recovery may occur after direct apposition [3]. When a tensionless direct suturing is not possible, nerve grafting should be considered [4, 5]. Recently, peripheral nerve defect is intended to reconstruct with allograft nerve collagen about substitutes for nerve autograft, because nerve grafts can cause sensory defects in the donor site [6]. There are various types of nerve allografts, for example, Gore-Tex and absorbable allograft tubes [7, 8]. In our case, we utilized the allograft nerve RENERVE®. Previously, meaningful animal experiment and clinical research were done clearly as follows.

Okamoto et al. studied a potential of RENERVE®. With 12 adult female beagle dogs weighting 10–12 kg, the peroneal nerve was cut to make a 30-mm defect. The nerve defect was bridged by RENERVE®. Comprehensive functional, electrophysiological, morphometrical, and histological analyses were performed for 1 year after the operation. The wet weight of the tibialis anterior muscles was only 32.4% of the healthy side at 24 weeks, which recovered to 77.4% 52 weeks after denervation. Electrophysiological evaluation of the tibialis anterior muscle belly showed a polyphasic wave 52 weeks after the implantation, which was almost half amplitude compared with that of the control. The results from this study showed the detailed process of morphological, electrophysiological, and functional recovery of the regenerated nerve, which would provide a scientific background for this novel therapy [9].

Saeki et al. made a clinical project to clarify the safety and efficacy of using RENERVE® in the treatment for nerve defects in humans. They conducted a multicenter, controlled, open-label study to compare the safety and efficacy of RENERVE® with those of the autologous nerve grafts. They included patients with sensory nerve defect of ≤ 30 mm, at the level of the wrist or a more distal location. They compared the sensory recovery using static two-point discrimination and adverse events between RENERVE® and autologous nerve grafting. As the result from these studies, the RENERVE® group included 49 patients, with a nerve defect of 12.6 mm. The autologous nerve graft group included 38 patients and nerve defect of 18.7 mm. The rate of recovery of sensory function at 12 months was 75% (36/49) for the RENERVE® group and 73.7% (28/38) in the autologous nerve group. No serious adverse events directly associated with the use of the RENERVE® were identified [10].

**Fig. 4:**
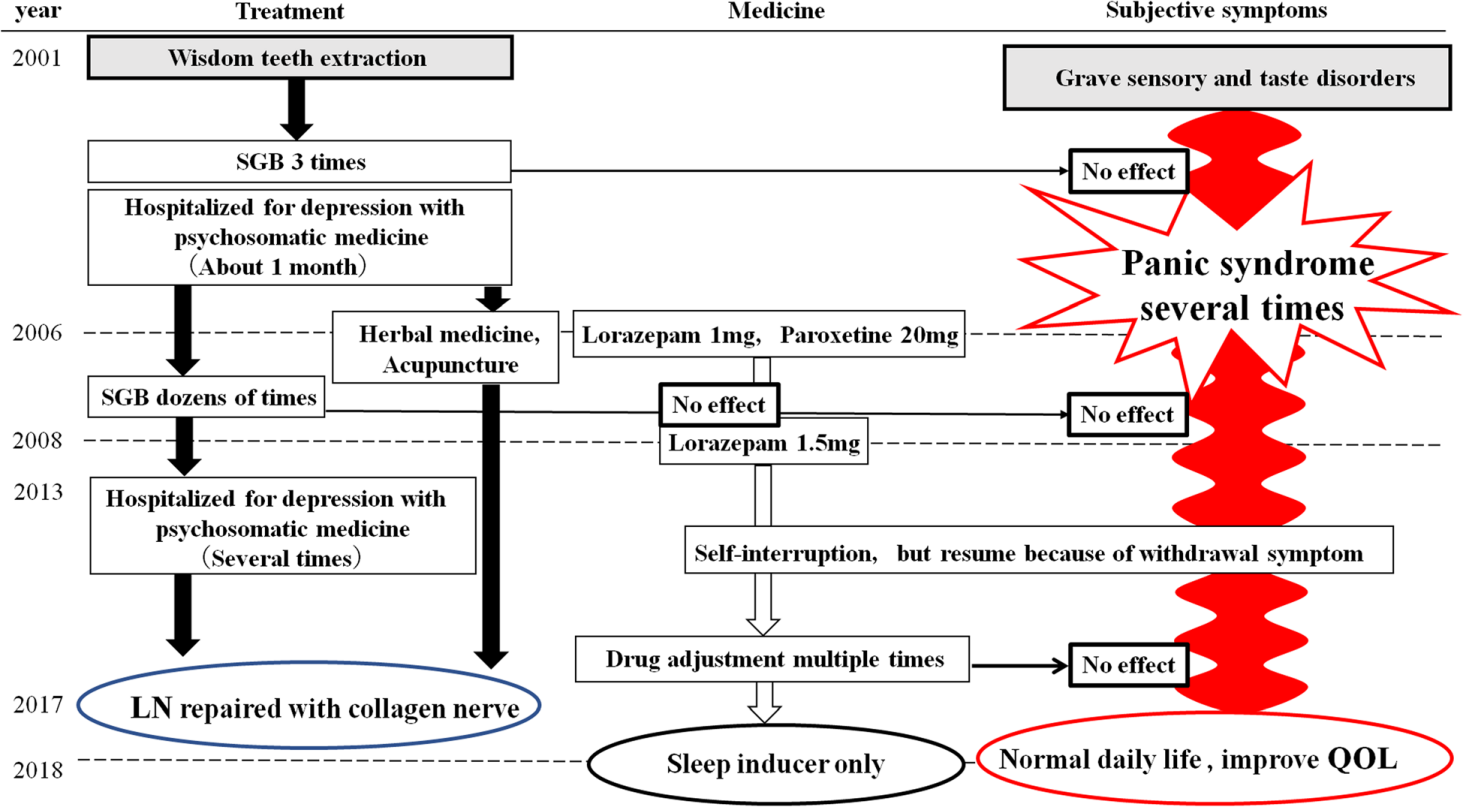
Prognosis. The patient obliged to take heavy psychotropic drugs for a long time before LN reconstruction. After the operation, he could be free from the heavy psychotropic drugs. Quality of her life was drastically refined after this operation

Time to repair played an important role in the overall surgical result, although the exact timing is still unclear. The patients with LN repair within 90 days of injury are said to have FSR within 1 year after repair in 93% of the cases [11]. Pogrel reported that microsurgical repair within 10 weeks of injury showed better results for FSR of the LN [12–14]. All the abovementioned opinions are in contrast with Robinson et al. Robinson et al. achieved a significant improvement in a number of sensory function categories including gustatory and functional results; however, they saw no correlation between time to repair and procedure success [15]. Minimally, we can agree with the opinion of Robinson et al. No consensus exists regarding the optimal methods and timing for disturbed lingual nerve repair. Our case had 17 years interval between the third molar teeth extraction and allograft collagen reconstruction. In spite of the extremely long span, we could get a precious result; There was recovery on gustatory sensation, 2PD, and SWT in the objective assessment, but unfortunately, in the subjective assessment, she is still angry with the first countermeasure of the dentist (Tables 1 and 2). Moreover, the important facts are the patient could be free from the heavy psychotropic drugs, which she continued to take in more than 7 years. The quality of her life was drastically refined after the operation (Fig. 4). the vein graft cuff method and the allograft collagen nerve (RENERVE®).

## Conclusion

In the early sick period, with the exact diagnosis like CNT, the LN disturbance should be managed. In a serious condition, the reconstruction with allograft nerve (RENERVE®) may be one of the recommendable methods.

## Data Availability

Not applicable.

## Abbreviations

2PD: Two-point discrimination
CNT: Clinical neurosensory testing
LN: Lingual nerve
MRCS: Medical Research Council Scale
SGB: Stellate ganglion block
SWM: Semmens-Weinstein monofilament
